# Innate, adaptive, and cell-autonomous immunity against *Toxoplasma gondii* infection

**DOI:** 10.1038/s12276-019-0353-9

**Published:** 2019-12-11

**Authors:** Miwa Sasai, Masahiro Yamamoto

**Affiliations:** 1 Department of Immunoparasitology, Research Institute for Microbial Diseases, Osaka University, Osaka, Japan; 2 Laboratory of Immunoparasitology, WPI Immunology Frontier Research Center, Osaka University, Osaka, Japan

**Keywords:** Parasitic infection, Infection

## Abstract

Hosts have been fighting pathogens throughout the evolution of all infectious diseases. *Toxoplasma gondii* is one of the most common infectious agents in humans but causes only opportunistic infection in healthy individuals. Similar to antimicrobial immunity against other organisms, the immune response against *T. gondii* activates innate immunity and in turn induces acquired immune responses. After activation of acquired immunity, host immune cells robustly produce the proinflammatory cytokine interferon-γ (IFN-γ), which activates a set of IFN-γ-inducible proteins, including GTPases. IFN-inducible GTPases are essential for cell-autonomous immunity and are specialized for effective clearance and growth inhibition of *T. gondii* by accumulating in parasitophorous vacuole membranes. Recent studies suggest that the cell-autonomous immune response plays a protective role in host defense against not only *T. gondii* but also various intracellular bacteria. Moreover, the negative regulatory mechanisms of such strong immune responses are also important for host survival after infection. In this review, we will discuss in detail recent advances in the understanding of host defenses against *T. gondii* and the roles played by cell-autonomous immune responses.

## Toxoplasmosis: Insights into immunity

Researchers are extensively studying immune responses to the single-celled parasite *Toxoplasma gondii*, which infects around one-third of humans, often harmlessly, but can cause life-threatening toxoplasmosis infections in patients with weakened immune systems. Masahiro Yamamoto and Miwa Sasai at Osaka University in Japan review recent advances in understanding the interactions between the immune system and the parasite. They consider non-specific ‘innate’ immune responses and also the ‘acquired’ responses that target specific parts of the parasite, referred to as antigens. Methods that selectively switch off genes in mice are revealing details presumed to also be relevant for humans. Significant molecules, molecular signaling pathways and immune-regulating processes are being identified. Recent studies suggest cell-autonomous immunity, the ability of host cells to defend themselves against attack, plays a significant role in fighting *Toxoplasma gondii* infection.

## Introduction

**Fig. 1:**
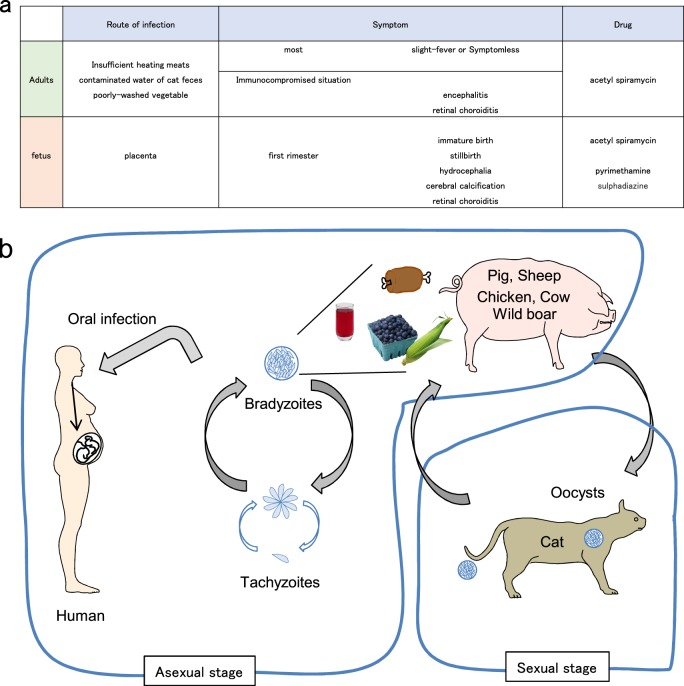
Toxoplasmosis and the life cycle of *T. gondii*. **a** Symptoms following *T. gondii* infection in humans. **b** Life cycle of *T. gondii*. When infected with *T. gondii*, felines shed oocysts in feces into the environment. The oocysts contaminate water, soil, fruits and vegetables from gardens and are easily taken in by humans and other intermediate hosts.

*Toxoplasma gondii* is a protozoan parasite belonging to the phylum Apicomplexa and can infect almost all warm-blooded animals. One-third of the human population is reported to be infected with *T. gondii*, rendering it classifiable as a common pathogen. Despite the high infection rate in humans, *T. gondii* infection is opportunistic in individuals with normal immunity. Conversely, in immunocompromised individuals, such as AIDS patients, those undergoing chemotherapy and immunosuppressed tissue transplant patients, infection and reactivation can lead to chronic infection with *T. gondii*, potentially resulting in lethal encephalitis (Fig. 1a)^1^. The life cycle of *T. gondii* is composed of two reproductive stages: sexual and asexual stages in definitive and intermediate hosts, respectively. *T. gondii* can establish sexual reproduction only in the epithelium of the digestive tract in cat species. On the other hand, the parasite asexually proliferates in all warm-blooded animals, including humans and livestock animals. Infection of intermediate hosts is mediated through oral digestion of cysts that are formed in tissues of intermediate hosts or oocysts that are formed in cat species. Oocysts or bradyzoites contained in the ingested cysts invade the host intestinal epithelium via the digestive tract wall and transform into tachyzoites. Subsequently, tachyzoites asexually proliferate several times and expand the infection in vivo. After the host detects *T. gondii* infection and activates the immune system, *T. gondii* tachyzoites transform into bradyzoites and form cysts in the central nervous or muscle tissues. The tissue cyst is a membranous structure containing lectins, such as N-acetylglucosamine and N-acetylgalactosamine, and can survive without being attacked by the host immune system (Fig. 1b)^2^. *T. gondii* actively infects host cells and resides in special membranous structures called parasitophorous vacuoles (PVs), where *T. gondii* is able to proliferate. If the PV is disrupted, *T. gondii* cannot survive within host cells. Host immune cells not only recognize *T. gondii*-derived ligands to activate acquired immunity but also destroy the PVs to remove proliferating *T. gondii* and ultimately block parasite expansion in vivo. However, *T. gondii* secretes various virulence factors into host cells to inhibit host acquired and innate immune responses. In this review, we will introduce recent findings regarding host defenses against *T. gondii* and the counter-defense mechanisms of the parasite.

## Innate and acquired immune responses against *T. gondii* infection

### Recognition of *T. gondii* by innate sensors

Innate immunity is the first line of host defense that responds immediately and detects pathogen infection via pattern recognition receptors (PRRs), such as Toll-like receptors (TLRs), Nod-like receptors, and C-type lectins. Ligand recognition by PRRs induces proinflammatory cytokines, including TNF-α, interleukin-6 (IL-6) and IL-12, and plays a role in the subsequent activation of immune responses^3–5^. The production of IL-12, which is important for the activation of acquired immunity in response to *T. gondii* infection, is severely impaired in MyD88-deficient mice^6^. MyD88 is an adaptor molecule that acts downstream of TLRs and the IL-1 receptor family members. TLRs are a group of 10 and 13 members in humans and mice, respectively, that differentially recognize pathogen-derived components and are involved in early pathogen detection. In mice, TLR11 has been shown to recognize *T. gondii*-derived profilin-like molecules, which are essential for parasite invasion of host cells. IL-12 is then induced in a MyD88-dependent manner^7,8^. Furthermore, TLR12 has been reported to detect profilin-like protein through its homodimer or as a heterodimer with TLR11, also resulting in IL-12 production^9^. In addition, IL-12 production in response to *T. gondii* infection has been shown to be reduced in mice that are deficient in the chemokine receptor CCR5. *T. gondii*-derived cyclophilin-18 (TgCyp18) is detected by CCR5 on dendritic cells (DCs), thus stimulating IL-12 expression^10^. CCR5 induces IL-12 production through activation of the G protein α I family and MAPK, suggesting that this CCR5-dependent signaling pathway plays an important role in the immune response against *T. gondii*^11,12^. Since CCR5 is mainly expressed on DCs, macrophages, T cells and microglia, CCR5-dependent production of inflammatory cytokines may be implicated in the induction of encephalitis. Moreover, given that CCR5-deficient mice show reduced embryonic lethality during pregnancy in comparison with that of wild-type mice, CCR5-dependent inflammation may be involved in fetal lethality and miscarriage when pregnant women undergo primary infection with *T. gondii*^13^. Both profilin-like protein and TgCyp18 stimulate IL-12 production by CD8α^+^ and CD8α^−^ DCs. Activation of TLR11 and TLR12 leads to IL-12 expression in plasmacytoid DCs. However, although *T. gondii* infection in mice causes IL-12 production in manners that are dependent on TLR11/TLR12 or CCR5, humans do not possess TLR11 and TLR12. How can humans produce IL-12 in response to *T. gondii* infection? Human monocytes produce IL-12 following *T. gondii* infection, suggesting that TLRs other than TLR11 and TLR12 may play a role in the detection of *T. gondii*-derived ligands in humans. TLR11 and TLR12 form heterodimers or homodimers that are expressed in endosomal compartments of the host cell cytosol. Since the ligand recognition mechanism of TLR11 and TLR12 in endosomes is similar to that of human TLR3, TLR7 and TLR9, which recognize nucleic acids, these TLRs, as well as TLR8, might be stimulated by *T. gondii*-derived nucleic acids in the endosome to induce IL-12 expression^14^. Although human CCR5 associates with TgCyp18, it is unclear whether CCR5 is critical for the production of IL-12 following *T. gondii* infection in humans^15^. Further investigation to assess the function of CCR5 on responses against *T. gondii* in humans will be required in the future. Furthermore, unidentified PRRs may be involved in the detection of *T. gondii* infection to produce IL-12 in a manner that is independent of MyD88 and CCR5. Future studies analyzing the machinery involved in *T. gondii* detection and IL-12 production in humans will be of interest. It has been very recently reported that human cells infected with *T. gondii* release S100A11 in a manner that is dependent on caspase-1, resulting in CCL2 production through receptor of AGE (RAGE) and monocyte chemotaxis to the infected site^16^. Thus, various factors released from infected cells strongly affect the subsequent activation of immune responses.

### Immune response at the infection site of *T. gondii*

*T. gondii* usually infects humans and animals through oral infection. When *T. gondii* cysts and oocytes present in undercooked meats or on unwashed fresh vegetables are ingested, the intestinal mucosa is the first place in the host body that encounters the organism. Innate lymphoid cells (ILCs) are a recently identified lymphocyte population consisting of three groups based on their functional characteristics^17^. Group 1 ILCs (ILC1s) consist of ILC1 and conventional natural killer (NK) cells. Activation of ILC1s leads to the production of Th1 cytokines, such as IFN-γ and TNF-α^18^. Group 2 ILCs (ILC2s) produce IL-4, IL-5, IL-9 and IL-13, which are Th2 cytokines. Group 3 ILCs (ILC3s) produce IL-17A and IL-22. When *T. gondii* infects orally, ILC1s produce IFN-γ and TNF-α in response to *T. gondii* infection^19,20^. In addition, the number of ILC3s in the lamina propria of aryl hydrocarbon receptor (Ahr)-deficient mice is decreased, resulting in overactivation of CD4 T cells in response to *T. gondii* infection. Thus, ILC3s and ILC1s are indirectly involved in the host defense against *T. gondii*^21^. Notably, the mechanism by which *T. gondii* infection activates ILCs is completely unknown. Moreover, the molecular mechanism by which ILC activation leads to the subsequent induction of acquired immune responses remains enigmatic at present.

### Acquired immune responses following sensing of *T. gondii*

The activation of DCs and macrophages in response to *T. gondii* infection results in the production of various cytokines, such as IL-1β and TNF-α^22–24^. Simultaneous administration of TNF-α and IL-1β or IL-1α in mice is protective against *T. gondii* infection in a manner that is highly dependent on IL-12^25^. IL-12 produced during innate immune responses in DCs, plasmacytoid DCs, and macrophages stimulates proliferation of NK cells, CD4^+^ T cells, and cytotoxic CD8^+^ T cells, resulting in massive IFN-γ production^26,27^ (Table 1). In particular, CD8α^+^ DCs migrating into local sites of *T. gondii* invasion detect the infection and produce IL-12, which further stimulates NK cells to generate IFN-γ in the bone marrow^28^. When severe combined immunodeficient (SCID) mice, which lack B and T lymphocytes, are infected with *T. gondii* and are treated with IL-12, systemic IFN-γ levels and eventual survival rates are increased, suggesting that IFN-γ derived from NK cells plays an important role in the host defense against *T. gondii* during the acute phase of *T. gondii* infection in vivo^29^. Even when NK cells or CD8^+^ T cells are depleted by antibodies in wild-type mice, acute resistance to *T. gondii* infection is unaffected^29^. In contrast, mice lacking the common γ-chain and are thus devoid of NK cells and CD8^+^ T cells, lose acute resistance to *T. gondii* in conjunction with further T cell depletion by anti-CD4 antibody, resulting in high susceptibility and indicating that IFN-γ produced by NK cells and CD4^+^ T cells contributes to the host defense against *T. gondii* host^29^. In addition, a recent study demonstrated that neutrophils can produce IFN-γ in TLR11-deficient mice that are systemically infected with *T. gondii*^30^. It is still unclear whether neutrophils produce IFN-γ in wild-type mice. Moreover, it has been very recently reported that a new cell population called memory-phenotype cells (MP cells), which are pathogen-independent memory-phenotype CD4 T cells, produce IFN-γ in response to IL-12 in *T. gondii* infection^31^. Thus, in addition to classical IFN-γ-producing cells, such as NK cells and T cells, new populations including ILC1s, neutrophils, and MP cells have been identified. However, the mechanism by which these classical and new IFN-γ-producing cells sequentially and mutually lead to IFN-γ production remains to be elucidated.

**Table 1:** List of major cytokines that are produced by *T. gondii* infection and the effect of each cytokine.

Cytokines	Producing cell	Effects	Refs.
IL-12	CD8α^+^or CD8αr CDCs	Activate and stimulate proliferation of NK cells, CD4 T cells and CD8 T cells	^6–8^
	Macrophages		
	pDCs		^9,10,25^
IFN-γ	ILC1s	Induce cell-autonomous immune responses	^19,20,24^
	CD4 T cells	iNOS induction	^26–28^
	CD8 T cells	IDO production	^29,32^
	NK cells		
	T-bet^+^Foxp3-Th1 cells		
TNF-α	Macrophages DCs	Induce other inflammatory proteins	^22–24^
IL-1β	Macrophages	Induce other inflammatory proteins	^22–24^
IL-10	Ly6ChighCCR2+ monocytes	Suppress inflammation to prevent toxoplasma encephalitis	^43,47,48^
	B cells	Suppress hyper inflammation	^49–51^
	T-bet+Foxp3-Th1 cells		^52–54^
IL-27	Myeloid cells	Required for resistance to chronic toxoplasmic encephalitis	^45,46,55^
		Induce CXCR3, T-bet, Blimp1 and IL-10 expression	
		Inhibit Th17 development	
IL-33	Damaged cells	Induce CCL2 expression (proinflammatory)	^56^
	Tissue injury	Induce IL-10 production by M2 macrophages (anti-inflammatory)	

Inducible nitric oxide synthase (iNOS) is an IFN-inducible protein that is also upregulated by *T. gondii* infection. Although lack of iNOS in mice does not affect acute control of *T. gondii* infection, iNOS-deficient mice are highly susceptible during the late phase of infection, resulting in lethality due to increased parasite burden in the brain^32,33^. Nitric oxide generated by iNOS activation plays a role in the suppression of cerebral toxoplasmosis in the fetus. Notably, the effect of iNOS can be detected in C57BL/6 but not in BALB/c mice, since *T. gondii* encephalitis does not usually occur in BALB/c mice^34^. In addition, IFN-γ also stimulates robust production of indoleamine 2,3-dioxygenase (IDO), which suppresses *T. gondii* growth in human fibroblasts, glioma cells, retinoblastoma cells, and macrophages^24,35–38^.

In addition to IL-12 production derived from DCs and macrophages, various types of host cells are involved in immune responses against *T. gondii*. When *T. gondii* infects a host, intestinal epithelial cells and peritoneal cells are primarily infected under physiological conditions. Antigen-presenting cells such as DCs and macrophages recognize *T. gondii*-derived factors, including virulence effectors, to produce chemokines such as CCL2 and CXCL2^39,40^. Such chemokines induce migration of Ly6C^high^CCR2^+^ monocytes and neutrophils to the sites of infection^41^. These monocytes and neutrophils that are infected with *T. gondii* spread systemically, while expression of IFN-γ and IFN-inducible proteins control the systemic infection^42^. However, Ly6C^high^CCR2^+^ monocytes also lead to the production of the anti-inflammatory cytokine IL-10, which is important for the suppression of *Toxoplasma* encephalitis, indicating that Ly6C^high^CCR2^+^ monocytes play both positive and negative roles in immune responses against *T. gondii*^43^. IL-10 produced by Foxp3^+^ CD4^+^ T cells (Tregs) is important for the maintenance of immune homeostasis in uninfected hosts. In contrast, the suppressive mechanism mediated by Tregs is not required during infection. Thus, the number of Tregs is tightly controlled. When *T. gondii* infects a host, local concentrations of IL-2 are decreased, resulting in the inhibition of Tregs and activation of the Th1 immune response^44^. Since Th1 cells are more highly responsive to IL-2 than Tregs, a local reduction in IL-2 is thought to selectively decrease the Treg population^44^ (Table 1). IL-27 and IL-12 synergize to limit IL-2 production during *T. gondii* infection^45,46^. However, immunosuppression after activation of Th1 cells is important for protection against excessive immunopathology in response to *T. gondii* infection. IL-10 and IL-22 belong to the IL-10 family of anti-inflammatory cytokines^47^. IL-10 is produced during not only the early phase post infection but also the chronic phase, especially in the brain^48,49^. IL-12 production is suppressed by Th2 cytokines, such as IL-4 and IL-10. Experiments using IL-10-deficient mice or wild-type mice treated with anti-IL-10 antibody demonstrate that IL-10 is produced by B cells and T-bet^+^Foxp3^−^ Th1 cells to suppress hyper inflammation^50–54^. T-bet^+^Foxp3^−^ Th1 cells produce IL-10 as well as IFN-γ, in a ‘self-regulation’ mechanism^54^. Moreover, IFN-γ stimulates T-bet expression in Tregs, subsequently leading to CXCR3 expression^55^. IL-27 stimulates not only T-bet and CXCR3 induction in Tregs but also IL-10 production. Blimp1 induces IL-10 to suppress inflammation and is also induced by IL-27^56^ (Table 1). IL-33 is also a proinflammatory cytokine that is induced by *T. gondii* infection and induces CCL2 expression in the brains of infected mice, leading to migration of CCR2^+^ monocytes and generation of the immune response against *T. gondii*. Conversely, IL-33 has been shown to amplify the Th2 response to suppress Th1 immunity and to stimulate IL-10 production by M2 macrophages to activate Tregs^57^. Thus, the role of IL-33 in the immune response against *T. gondii* has two opposing aspects and hence requires further investigation to fully elucidate the molecular mechanisms involved (Table 1).

## Cell-autonomous immune responses against *T. gondii* infection

### IRG-mediated cell-autonomous immune responses

**Fig. 2:**
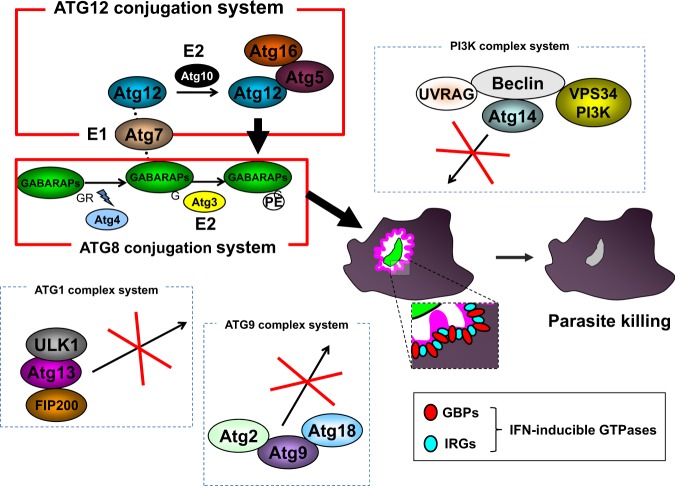
Diagrams of cell-autonomous immune responses to *T. gondii* infection. IFN-inducible GTPases, such as GBPs and IRGs, accumulate on the *T. gondii* PVM and are important for its disruption. ATG family proteins are functionally divided into five systems, Atg12 conjugation, Atg8 modification, Atg1 complex, PI3K complex and Atg9 complex, which all play differentially critical roles in autophagy. Among them, IFN-inducible GTPase-dependent immunity requires Atg12 conjugation and Atg8 modification systems (marked red). Atg family members (marked red) regulate the localization of the GTPases that are involved in the destruction of PVMs. GTPases that are reportedly recruited to PVM are IRGs (Irga6, Irgb6, Irgb10 and IrgD) and GBPs (GBP1, GBP2, GBP3, GBP5, GBP6, GBP7 and GBP9). Some GTPases on the PVM form pores on the PVM, resulting in destroying the replication location of *T. gondii* and preventing the expansion of infection.

IFN-γ produced by activated immune cells such as NK cells, ILC1 and T cells has been shown to play a pivotal role in anti-*T. gondii* immunity^58^. IFN-γ-stimulated cells express hundreds of IFN-stimulated genes, including GTPase family members. Among them, a recent extensive analysis of p47 and p65 GTPases revealed that these GTPases are strongly involved in the immune response against *T. gondii*^59^. Immunity-related GTPases (IRGs) (also called p47 GTPase) are family members whose molecular weights are ~47–48 kDa. IRGs consist of IRGM, IRGA and IRGB family members, all of which possess GTP-binding domains, whose amino acid sequences can be classified into two major groups, GMS-IRGs and GKS-IRGs^60,61^. GMS-IRGs consist of IRGM1^62^, IRGM2^60^ and IRGM3^63^, and are mainly localized in the Golgi and ER^64–66^. The remaining IRGs, excluding the GMS-IRGs, are classified as GSK-IRGs, among which IRGA6^60^ and IRGB6^67,68^ are strongly involved in the immune response against *T. gondii*^61^. The mechanism by which GMS-IRGs regulate GKS-IRGs remains unclear. GMS-IRGs form heterodimers with GKS-IRGs to negatively regulate activation^69^. Regarding GKS-IRGs, it has been reported that IRGB10 and IRGD are recruited to the PVM following *T. gondii* infection, but it is not clear if they are critical in preventing *T. gondii* infection in vivo^70^. Moreover, it remains unclear whether GTPases are involved in responses against *T. gondii* and which are not involved because many GTPases are induced. Cells from mice lacking IRGM1 or/and IRGM3 showed decreased IRGA6 and IRGB6 protein expression levels and abnormal localization, resulting in impaired immune response against *T. gondii*^71,72^. PVs are specialized compartments generated by *T. gondii* in infected host cells. *T. gondii* resides within the PV and proliferates until transmission to the next host cells^59,73^. Among the IRGs, IRGA6, and IRGB6 in particular accumulate on the PV membrane (PVM) and destroy this structure^69,74^ (Fig. 2).

P65 GTPases are called guanylate-binding proteins (GBPs) and also play important roles in the host defense against *T. gondii*. Humans and mice possess 6 and 11 GBPs, respectively. In mice, GBPs form two gene clusters located on chromosomes 3 and 5 encoding GBP^*chr3*^ and GBP^*chr5*^, respectively^75^. GBP^*chr3*^ consists of GBP1, GBP2, GBP3, GBP5 and GBP7. GBP1 and GBP7 have been shown to be involved in host defense against not only *T. gondii* but also *Listeria monocytogenes* and *Mycobacterium bovis* BCG^76,77^. Mice lacking GBP^chr3^ are severely defective in the destruction of the *T. gondii* PVM in macrophages and exhibit dramatically high susceptibility to the pathogen^78^. In contrast, mice singly lacking GBP1 or GBP2 demonstrate a milder phenotype than that of GBP^*chr3*^-deficient mice, indicating that GBP^*chr3*^ members other than GBP1 and GBP2 play a role in immune responses against *T. gondii* by compensating for the loss of GBP1 and GBP2^77,79^ (Fig. 2). The precise functions of other GBPs such as GBP3 and GBP5 that are recruited to the PVM need to be clarified in the future.

### Ubiquitination of PVMs by IFN stimulation

Although it is well documented that IFN-inducible GTPases play an important role in the immune response against *T. gondii*, the mechanism by which these GTPases are recruited to the PVM remains unclear. Analysis using a chimeric protein of GBP2 and GBP5 revealed that isoprenylation of GBP2 at the C-terminus is essential for GBP2 recruitment to the PVM. However, C-terminal isoprenylation of GBP2 is not sufficient to specifically recognize the PVM^80^. Given that host recognition of cytosolic bacteria involves ubiquitination of bacteria or bacteria-containing vacuoles, ubiquitination might be one way to distinguish host and *T. gondii* PVMs^81,82^. To date, modes of ubiquitination of *T. gondii* PVMs have been shown to be K48-linked, K63-linked and M1 linear types^83,84^. Analysis using cells lacking E3 ligases such as TRAF6 or TRIM21 demonstrated that these E3 ligases are involved in IFN-γ-induced ubiquitination of *T. gondii* PVMs^85^. However, given that the degree of ubiquitination and PV destruction in TRIM21-deficient cells is minimal, other E3 ligases, including TRAF6, may compensate for the loss of TRIM21. Analysis using cells with compound mutations in TRIM21 and TRAF6 will clarify whether these proteins compensate for each other or if other E3 ligases are involved. Recently, it has been reported that murine GBP1 and GBP2, which are members of the p65 GTPases, are ubiquitinated by TRIM21^86^. However, the biological effects of ubiquitination of GBPs are completely unknown. Further research will clarify the function of ubiquitination following *T. gondii* infection.

In addition to the molecular mechanism by which IFN-inducible GTPases are recruited to the *T. gondii* PVM, the biological significance of the recruitment of the ubiquitin-binding protein p62 to the PVM remains controversial. Similar to IFN-inducible GTPases, p62 plays an important role in *T. gondii* clearance^87^. Conversely, another report demonstrated that p62 is not required for IFN-γ-induced *T. gondii* clearance but is involved in the presentation of antigens located in the PV to activate CD8^+^ T cells^83^.

### Regulation of cell-autonomous immune responses by autophagy-related genes

Autophagy is a highly conserved biological response and is required for the maintenance of homeostasis by self-digestion during periods of stress, such as nutrient starvation or embryonic development^88^. Autophagy is strictly regulated by autophagy genes and their products^88^. The mammalian homolog of ATG8 consists of the LC3 and GABARAP subfamilies. The LC3 subfamily comprises LC3A, LC3B and LC3C. In addition, the GABARAP subfamily consists of GABARAP, GABARAPL1 and GABARAPL2 (also known as Gate-16)^89^. LC3B is a homolog of yeast ATG8 and accumulates on the autophagosome to remove cytosolic pathogens. Subsequently, LC3B-coated autophagosomes fuse with the lysosome to digest the pathogens^90^. This particular process of autophagy is called xenophagy^91^. When cells are activated by CD40, LC3B also accumulates on *T. gondii* PVMs. Stimulation with CD40 induces physical interaction of the PVM and the lysosome, possibly leading to activation of PI3K3C, Rab7, and vATPase^92^. However, the recruitment of LC3B to the *T. gondii* PVM is controversial^93^. LC3B might be involved in the functionality of the IFN-γ-inducible anti-*T. gondii* response.

During autophagy, ATG5 and ATG12 form a complex to mediate the modification of phosphatidylethanolamine on the C-terminus of ATG8^94^. Cells lacking ATG5, an essential autophagy protein, are also severely defective in IFN-γ-induced PVM destruction, which is essential for the inhibition of *T. gondii* proliferation^95^. In general, although the autophagosome is a double-membraned structure, the *T. gondii* PV does not possess a double membrane, suggesting that the function of ATG5 in the immune response against *T. gondii* is independent of autophagy^96^. Subsequently, the GABARAP subfamily but not the LC3 subfamily plays an important role in the uniform distribution of GBPs in host cells to efficiently mediate host defense against *T. gondii*. GBPs are located on small vesicle-like structures (VLSs)^77^. Among GABARAP subfamily members, Gate-16 in particular is involved in the uniform localization of GBP2-containing VLSs. GBP2-containing VLSs aggregate in the cytosol and are dysfunctional in Gate-16-deficient cells. Mice lacking Gate-16 specifically in the myeloid cell lineage are defective in GBP-dependent host defense against *T. gondii* in vivo^42^ (Fig. 2). Even Gate-16-deficient or Atg5-deficient cells showed dramatically increased *T. gondii* numbers, and parasite numbers in autophagy-deficient cells, such as cells lacking FIP200 and Atg9, were intact following IFN-γ-stimulation. The effects of xenophagy on immune responses against *T. gondii* are not extensive.

## Conclusion

**Fig. 3:**
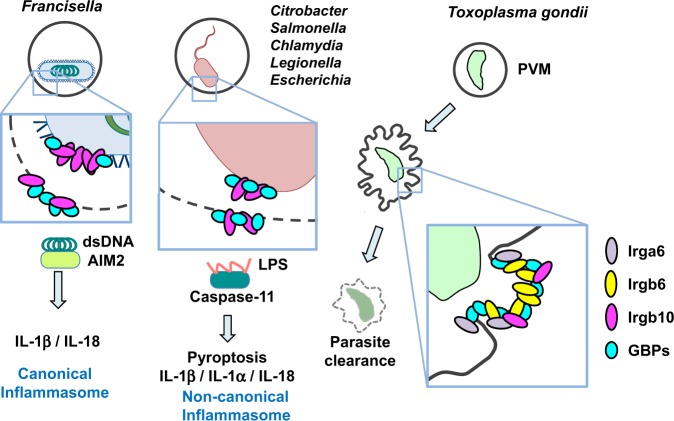
IFN-inducible GTPase-dependent cell-autonomous immunity to intracellular pathogens. *Francisella* is Gram-negative bacterium that resides within a phagosome called the *Francisella*-containing phagosome (FCP), which is an initial vacuolar compartment. DNA from *Francisella* is somehow released into the cytosol and is recognized by AIM2, which is a cytosolic sensor that recognizes DNA, following the activation of the canonical inflammasome. Some bacteria, such as *Salmonella* and *Citrobacter*, are also Gram-negative bacteria and remain in the vacuoles in the host cytosol. LPS from these bacteria somehow binds to caspase-11 and then activates noncanonical inflammasomes. IRGs and GBPs accumulate on PVs of *T. gondii*, bacteria-containing vacuoles or bacterial cell membranes of various intracellular bacteria to eliminate pathogens and subsequently induce activation of the inflammasome. The function of each IRG and GBP on vacuoles is not fully understood.

Innate and adaptive immune responses to *T. gondii* are being extensively studied using gene knockout mice. Among these analyses, cell-autonomous immunity involving IFN-γ-inducible GTPases, such as GBP and IRG provides us with a new concept that is distinct from previously established immunological ideas. Recently, cell-autonomous immunity has been shown to have a defensive function against pathogens other than *T. gondii* (Fig. 3). GBP1 is reported to be involved in the suppression of *L. monocytogenes* or *M. bovis* BCG infection^76^. GBP2 and IRGB10, another IRG member, accumulate on chlamydia-containing vacuoles in a manner that is dependent on Atg3 and Atg5^97^. In addition, GBP^chr3^ is involved in LPS-induced caspase-11 activation that is induced by infection with *Legionella*, *Salmonella* or *E. coli* to inhibit bacterial growth by a mechanism of cell death known as pyroptosis^98^. Moreover, *Chlamydia* infection causes activation of the canonical inflammasome involving caspase-1 as well as the caspase-11-dependent noncanonical inflammasome in a GBP-dependent manner^99^. Furthermore, GBPs induced by IRF1 are important for AIM2-dependent detection of double-stranded DNA resulting from cytosolic *Francisella* proliferation^100,101^ (Fig. 3). IRGB10 is also involved in GBP^chr3^-dependent AIM2-induced activation of the inflammasome^102^. GBPs are required for noncanonical inflammasome activation mediated by outer membrane vesicles, which are released from Gram-negative bacteria and contain LPS^103^ (Fig. 3). Thus, given that GBPs and IRGs play various roles in regulating host defense and pathogen infection, it is reasonable to assume that pathogens establish counter-defense mechanisms. For instance, *Shigella* possesses an effector molecule called IpaH9.8 to inhibit GBP2-dependent immune responses against *Shigella*^104,105^. As such, the pathogen-mediated anti-“anti-pathogen immune response” should be further analyzed. In particular, the exact molecular mechanisms by which GBPs and IRGs mediate cell-autonomous immunity against *T. gondii* and other pathogens, including bacteria and viruses, will be of interest in the future.
